# One-Step Formation of Silicon-Graphene Composites from Silicon Sludge Waste and Graphene Oxide via Aerosol Process for Lithium Ion Batteries

**DOI:** 10.1038/srep33688

**Published:** 2016-09-20

**Authors:** Sun Kyung Kim, Hyekyoung Kim, Hankwon Chang, Bong-Gyoo Cho, Jiaxing Huang, Hyundong Yoo, Hansu Kim, Hee Dong Jang

**Affiliations:** 1 Rare Metals Research Center, Korea Institute of Geoscience & Mineral Resources, Daejeon, 34132, Korea; 2 Department of Nanomaterials Science and Engineering, University of Science & Technology, Daejeon, 34113, Korea; 3 R& D Center for Valuable Recycling, Korea Institute of Geoscience & Mineral Resources, Daejeon, 34132, Korea; 4 Department of Materials Science and Engineering, Northwestern University, Evanston, Illinois 60208, USA; 5 Department of Energy Engineering, Hanyang University, Seoul, 04763, Korea

## Abstract

Over 40% of high-purity silicon (Si) is consumed as sludge waste consisting of Si, silicon carbide (SiC) particles and metal impurities from the fragments of cutting wire mixed in ethylene glycol based cutting fluid during Si wafer slicing in semiconductor fabrication. Recovery of Si from the waste Si sludge has been a great concern because Si particles are promising high-capacity anode materials for Li ion batteries. In this study, we report a novel one-step aerosol process that not only extracts Si particles but also generates Si-graphene (GR) composites from the colloidal mixture of waste Si sludge and graphene oxide (GO) at the same time by ultrasonic atomization-assisted spray pyrolysis. This process supports many advantages such as eco-friendly, low-energy, rapid, and simple method for forming Si-GR composite. The morphology of the as-formed Si-GR composites looked like a crumpled paper ball and the average size of the composites varied from 0.6 to 0.8 μm with variation of the process variables. The electrochemical performance was then conducted with the Si-GR composites for Lithium Ion Batteries (LIBs). The Si-GR composites exhibited very high performance as Li ion battery anodes in terms of capacity, cycling stability, and Coulombic efficiency.

Silicon (Si) has been widely used as an important base material in the semiconductor, photovoltaic (PV), and microelectronics industries^1^,^2^. However, more than 40% of the Si material goes into waste sludge during the slicing of silicon into ingot wafers, which causes a severe loss of valuable resources as well as environmental contamination^3^,^4^. Recently, recovery of Si particles from waste silicon sludge has been a great concern due to the demand for the recycling of valuable high-purity silicon, for improved environmental protection, and for many potential applications as value-added materials^5^,^6^. In order to develop recycling technology that can convert waste silicon sludge into value-added materials, the recovery of Si from sludge represents a first step. A few studies have been conducted to recover Si particles from waste silicon sludge using different methods^7^,^8^. Lin *et al*.^3^ reported a Si-recovery process from waste silicon sludge that involved the removal of impurities by an acid treatment followed by multi-stage centrifugation^7^. Zhang *et al*.^8^ devised a filtration method that used foam filters at high temperatures to recover Si metal^8^. In our previous work, we showed an effective process to recover pure Si nanoparticles by sonication and centrifugation after impurity removal^9^. We then also developed a convenient one-step method to recover Si particles by an ultrasonic atomization-assisted aerosol process^10^.

One of the very promising applications of recovered Si particles for value added materials is as anode materials in lithium ion batteries (LIBs) because Si exhibits the highest theoretical energy density of 3579 mAh/g (based on Li_15_Si_4_) among anode materials for LIBs^11^,^12^. However, although Si is regarded as one of the most promising anode materials for LIBs, some technical problems need to be solved for commercial use of Si for LIBs. One of the main problems of Si is its large volume fluctuation with a repetitive lithiation/delithiation process, which leads to pulverization of the Si particles, which in turn causes fast capacity fading during cycling^13^. Another problem is that a solid electrolyte interface (SEI) film will be repeatedly formed at each cycle because of the exposure to the newly created fresh surface of the Si associated with the repeated volume changes during cycling^14^,^15^. These two restrictions create low coulombic efficiency and poor cycling stability of the Si anode electrode. Many efforts have been undertaken to address these problems, such as applying carbon materials on Si particles^16^,^17^. Among carbon materials, graphene (GR) has gained much attention for energy storage applications due to its prominent features, such as high surface area, superior mechanical flexibility, excellent electronic conductivity and fast charge carrier mobility^18^. The GR can also act as an electrolyte-blocking layer to control the SEI formation on the surface of Si and a buffer layer to alleviate the volume changes of Si during cycling^19^. The Si-GR hybrid materials are expected to improve the electrochemical performances of Si: acceptable volume change of Si particles and high electrical conductivity in the presence of the GR matrix. Recently, a few studies were conducted to produce Si-GR composites using commercial Si nanoparticles for LIBs using chemical reduction in a liquid-phase reaction, steam etching, and ball milling^14^,^20^,^21^. Yi *et al*.^20^ reported GR-wrapped Si-C composite by chemical process such as heating, etching, and carbon coating. It shows acceptable reversible capacity of approximately 1200 mAh/g and high areal capacity of 3.2 mAh/cm^2^ after 100 cycles^20^. Tang *et al*.^21^ synthesized a self-assembly Si/porous reduced graphene oxide composite film by chemical reduction in a liquid-phase reaction, which show good specific capacity and cycling stability (1261 mAh/g)^21^. Although all Si-GR hybrid materials show the good electrochemical performance, the previous studies are required much time to prepare the Si-GR composites and used the expensive Si nanoparticles. Thus, a more advanced process to synthesis of Si-GR hybrid materials from Si sludge with a low price, a short operating time and an eco-friendly process is greatly required.

In our previous work, we fabricated GR encapsulated Si nanoparticles via spray drying and thermal treatment, which showed a stable capacity of 940 mAh/g^19^. All Si-GR hybrid materials showed good electrochemical performance, which could be attributed to the fact that GR could not only enhance the electrical conductivity of Si but also effectively release the volume expansion and aggregation of Si nanoparticles during the charge/discharge processes^19^.

Since the commercial Si nanoparticles for LIB applications are still suffering from their high cost, we then developed an aerosol-assisted method to extract Si particles of less than 200 nm from Si sludge wastes^9^. We then synthesized a GR encapsulated Si composite again via aerosol process^10^. The GR encapsulated Si fabricated from the extracted Si particles had a high reversible capacity of about 1750 mAh/g with superior capacity retention of the pure Si nanoparticles^10^. Although this process was quite successful, it required two steps in order to extract Si particles from Si sludge waste and synthesize the GR-encapsulated Si composite. Thus, we considered what was necessary was a more advanced process to obtain the GR encapsulated Si composite with low energy consumption, a short operating time and an improved recovery rate.

In this paper, we first introduce a novel one-step process which not only extracts Si particles but also generates Si-GR composites from a colloidal mixture of waste Si sludge and graphene oxide (GO) at the same time by ultrasonic atomization-assisted spray pyrolysis. This aerosol process supports lower energy consumption, a shorter operating time and an improved recovery rate compared to previous results for forming GR encapsulated Si composites from a colloidal mixture of Si, SiC and GO. We examine the effects of the operating temperature, Si concentration and graphene oxide (GO) concentration on the particle properties, i.e., the morphology and crystallinity. The electrochemical performance of the as-prepared Si-GR composites was investigated as anode materials for LIBs in terms of capacity, cycling stability, and Coulombic efficiency.

## Results

### Formation of Si-GR composite

**Figure 1:**
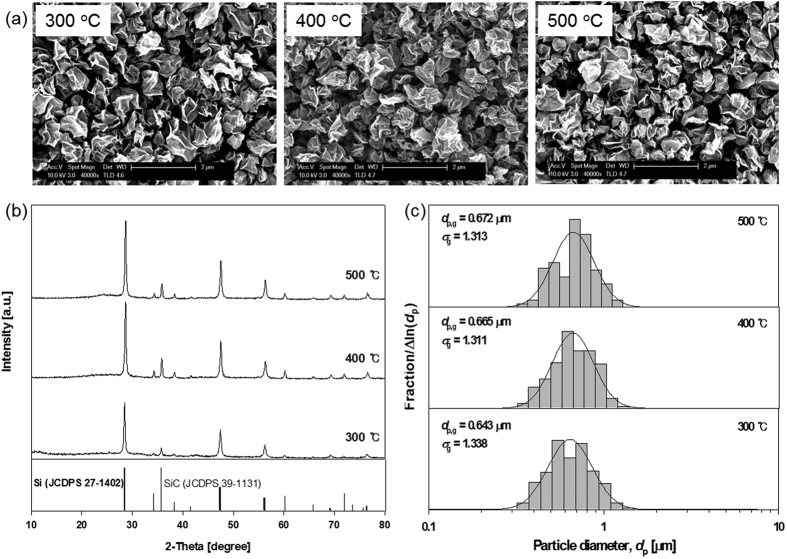
Morphology (**a**), X-ray diffraction pattern (**b**), and particle size distribution (**c**) of the Si-GR composites prepared at different operating temperatures of 300 °C, 400 °C, and 500 °C (GO: 0.1 wt%, Si sludge: 1.0 wt%).

Figure 1 shows the morphology, X-ray diffraction patterns, and particle size distribution of the as-prepared Si-GR composites with respect to the operating temperature from 300 to 500 °C. The FE-SEM analysis indicated that the as-prepared Si-GR composites had a crumpled paper ball-like morphology and the Si particles were completely encapsulated by GR sheets (Fig. 1a, Supplementary materials Figure S1). There were no differences in the morphology according to the operating temperature in the FE-SEM analysis. The XRD analysis showed that the composites consisted of Si and SiC, while GR was not clearly shown because the intensity of the GR phase was much lower than that of the Si and SiC phases, while the Si, SiC, and GR references were at 28, 35 and around 25 degrees, respectively (Fig. 1b). The extracted Si particles had a higher intensity at a higher operating temperature. It was found that 78 wt% of Si was recovered from the Si sludges by the aerosol process. The mass fraction of SiC in the recovered material was 3.2 wt% by a chemical measurement method^22^. Although a little SiC still remained in the extracted particles, the Si particles were effectively extracted by the one-step aerosol process. Also, the residual SiC may contribute the long-term cyclability and high conductivity in electrochemical properties^23^. As the operating temperature increased from 300 to 500 °C, the average size of the composite remained almost the same at about 0.66 μm (Fig. 1c). The size distribution of the composites was quite uniform and the geometric standard deviation was about 1.3. In the absence of GO, Si agglomerates recovered by aerosol process were spherical and 0.47 μm in average diameter (Supplementary materials Figure S2).

**Figure 2:**
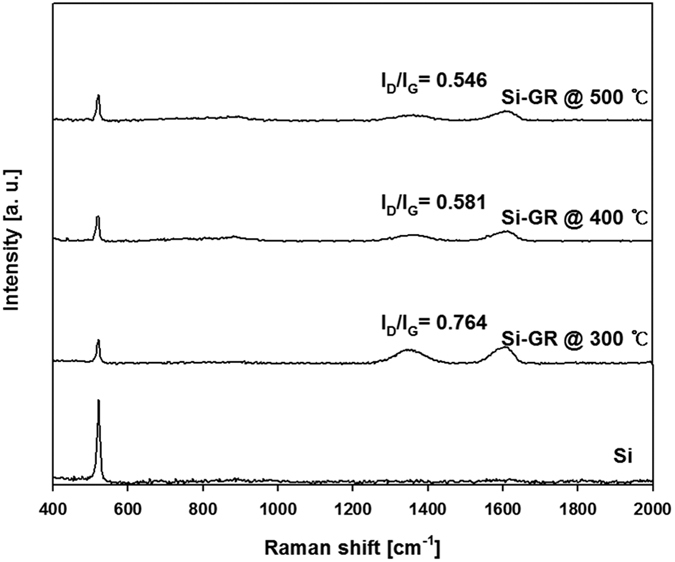
Raman spectrum of the Si-GR composites prepared at different operating temperatures of 300 °C, 400 °C, and 500 °C (GO: 0.1 wt%, Si sludge: 1.0 wt%).

Figure 2 shows the Raman spectra of the as-prepared Si-GR composites along with the operating temperature. There were Si peaks in the Si-GR composites at 519 cm^−1^ and also a remarkable peak of GR at 1600 cm^−1^ in the G band and 1350 cm^−1^ in the D band. The D band, the A_1g_ breathing mode, is associated with the extent of the defects in the curved graphite sheet, sp^3^ carbon, or other impurities. The G band exhibited first-order scattering of the E_2g_ mode in the sp^2^ carbon domains, corresponding to the opposite direction of movement between two neighboring carbon atoms^24^. As the operating temperature increased from 300 to 500 °C, the I_D_/I_G_ ratio decreased from 0.76 to 0.55. We attributed this to the increased reducing rate from GO to GR and the decreasing number of defects in the GO during the removal of oxygen at higher temperature. In addition, the specific surface area of the Si-GR composite increased from 28 to 54 m^2^/g as the operating temperature increased (Table 1).

**Figure 3:**
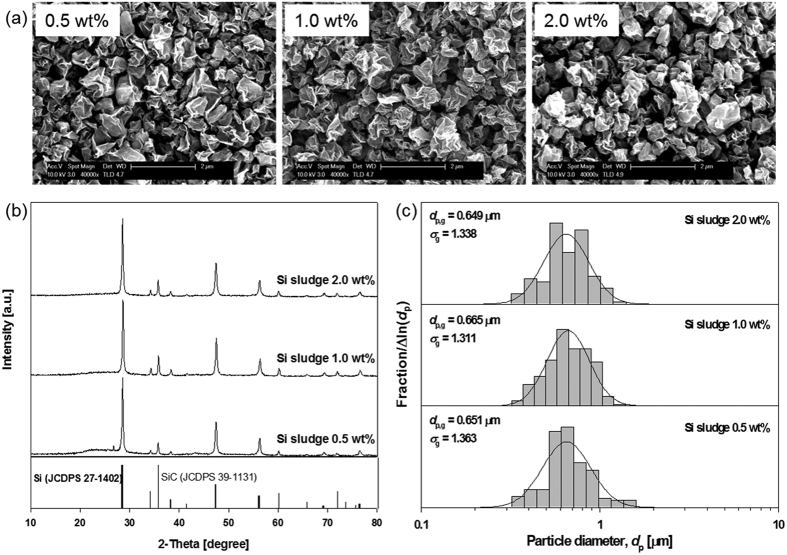
Morphology (**a**), X-ray diffraction pattern (**b**), and particle size distribution (**c**) of the Si-GR composites prepared at different Si sludge concentrations of 0.5 wt%, 1.0 wt%, and 2.0 wt% (GO: 0.1 wt%, operating temperature: 400 °C).

Figure 3 shows the morphology, X-ray diffraction patterns, and particle size distribution of the as-prepared Si-GR composites at different concentrations of Si sludge in the colloidal mixture from 0.5 to 2.0 wt%. There are no large point of differences in the morphology diffraction patterns and particle size distribution with respect to the concentration of Si. In the XRD result, the GR phase is observed at around 25 degrees in only a 0.5 wt% concentration of Si sludge (Fig. 3b). This is attributed to the relatively higher concentration of GO at a 0.5 wt% concentration of Si sludge than other conditions. The specific surface area of the Si-GR composite decreased from 64 to 35 m^2^/g according to concentrations of Si sludge in the colloidal mixture from 0.5 to 2.0 wt%.

**Figure 4:**
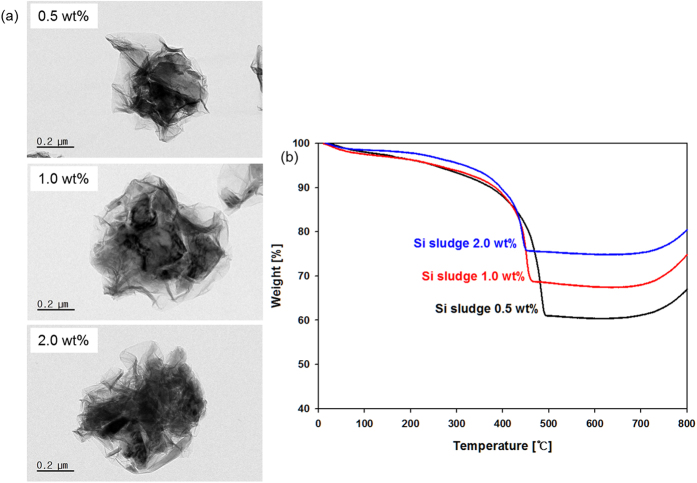
TEM images (**a**) and TGA curves (**b**) of the Si-GR composites prepared at different Si sludge concentrations of 0.5 wt%, 1.0 wt%, and 2.0 wt% (GO: 0.1 wt%, operating temperature: 400 °C).

The TEM images make it clear that Si particles were encapsulated by GR sheets (Fig. 4a). They also indicate that larger agglomerates consisting of Si particles were located inside the GR shell at higher Si sludge concentrations. Figure 4b shows the TGA curves according to the Si sludge concentration. To determine the content of Si in the Si-GR composites, TGA was performed in a temperature range from 25 to 800 °C with a heating rate of 5 °C/min in air.

According to the TGA analysis, the mass fractions of Si with increasing Si sludge concentrations were about 61, 69, and 76%, respectively. This result thus confirms that the Si/GR ratios of the as-prepared samples are consistent with the pre-determined Si/GR ratio of the aerosol precursor, and that the aerosol process is a very efficient tool for controlling the composition of Si particle encapsulated GR composite materials. Further, the weight curves of all the samples slightly increased above 700 °C due to the oxidation of Si particles. We believe that the morphology of the GR encapsulated Si composite protects Si particles against SEI film formation, which results in improved electrochemical performance.

**Figure 5:**
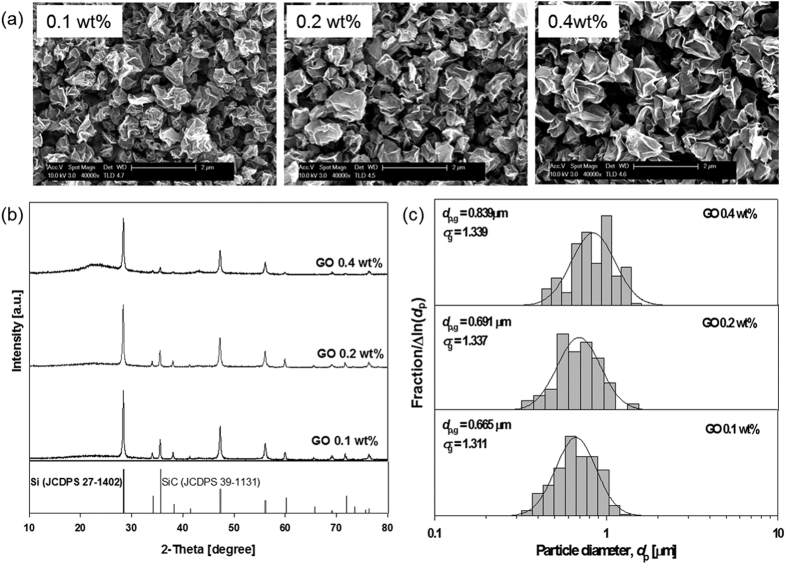
Morphology (**a**), X-ray diffraction pattern (**b**), and particle size distribution (**c**) of the Si-GR composites prepared at different GO concentrations of 0.1 wt%, 0.2 wt% and 0.4 wt% (Si sludge: 1.0 wt%, operating temperature: 400 °C).

Figure 5a,c show the morphology, and particle size distribution of the as-fabricated Si-GR composites according to the concentrations of GO in the colloidal mixture. As the GO concentration increased from 0.1 to 0.4 wt%, the average size of the composite slightly increased from 0.67 to 0.84 μm. When GO concentration is getting higher in the colloidal mixture, higher numbers of GR sheets exist in a single sprayed droplet, so that they became more stacking between the GR sheets in the Si-GR composites. The XRD patterns of the as-prepared Si-GR according to the GO concentration are shown in Fig. 5b. The intensity of GR of the composites increased as GO concentration increased from 0.1 to 0.4 wt% (Supplementary materials Figure S3). The specific surface area of the Si-GR composite increased from 37 to 52 m^2^/g with respect to GO concentration.

### Lithium ion storage characteristics of Si-GR composites

**Figure 6:**
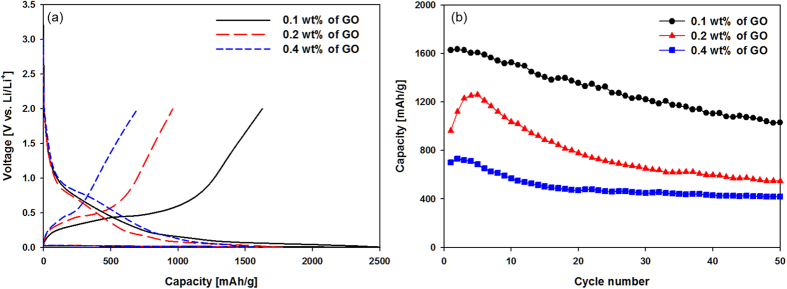
(**a**) Voltage profiles of Si-GR nanocomposite electrodes for the first cycle and (**b**) Cycle performance of Si-GR nanocomposite electrodes with respect to the concentration of GR added in the composites.

Figure 6a shows the voltage profiles of Si-GR composite electrodes at a rate of 0.2 C (200 mA/g) with respect to the concentration of GO in the colloidal mixture for the first cycle. The charge capacity (lithium ions extraction) of Si-GR electrodes was 1626 mAh/g for Si-GR composite with 0.1 wt% of GO, 960 mAh/g for the sample with 0.2 wt% of GO and 700 mAh/g for the sample with 0.4 wt% of GO, respectively (Supplementary materials Figure S4). This tendency, the decrease of the capacity with respect to the amount of GO can be ascribed to the presence of GR shell, which acted as a lithium ion barrier, thus decreasing the discharge capacity. Si-GR composite with 0.2 wt% of GO electrode showed the increase of the capacity with the increase of the cycle numbers during the initial few cycles. This tendency might be attributed to the volume expansion and contraction of Si-GR composite during cycling. This repeated volume changes of Si-GR composite led to the exposure of Si to the electrolyte, thereby showing the increase of the capacity. The initial Coulombic efficiencies of Si-GR composite electrodes had the same tendency; the Si-GR nanocomposite with the smallest GR showed the highest initial Coulombic efficiency of 63.89% compared with the Si-GR composite with a higher amount of GR added (54.07% for the sample with 0.2 wt% of GO concentration and 44.29% for the sample with 0.4 wt% of GO concentration). This capacity can be explained by the presence of GR in the composite because highly conductive GR in the composite might increase the amount of the electrolyte decomposition reaction to form a SEI on the surface of the electrode, thereby decreasing the Coulombic efficiency of the Si-GR composite electrode for the first cycle. Figure 6b shows the capacity retention of Si-GR composite electrodes at a rate of 0.2 C (200 mA/g) during 50 cycles. On the contrary to the discharge capacity and the initial Coulombic efficiency of Si-GR nanocomposite electrodes as shown in Fig. 6b, the Si-GR composite electrode with a higher content of GR had a much improved cycle performance over those with a lower content of GR added in the composite. This relationship between the cycle performance and the relative amount of GR in the Si-GR composite is mainly because, as we mentioned previously, the GR in the composite plays a key role in alleviating the mechanical strains induced by the huge volume changes in Si during cycling.

**Figure 7:**
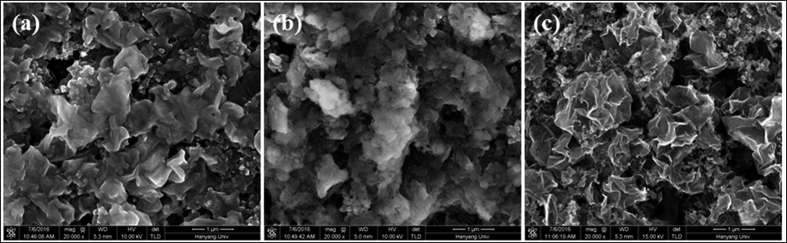
Top view FE-SEM image of Si-GR composites electrode (**a**) pristine, (**b**) after the first charge (lithiation) and (**c**) after 1 cycle.

Figure 7 shows FE-SEM images of pristine Si-GR composite electrode, Si-GR composite electrode after fully lithiation and Si-GR composite electrode after 1 cycle. As shown in Fig. 7, FE-SEM image of Si-GR composite materials after 1 cycle was almost same to that of pristine Si-GR composite materials. This comparison indicates that the Si-GR composite electrode showed reversible morphological changes during cycling, which might be attributed that the inner free spaces between the GR shell and Si nanoparticles acted as a mechanical buffer to accommodate the huge volume changes of Si nanoparticles during cycling.

## Discussion

One-step formation of Si-GR composites from a colloidal mixture of Si sludge and GO was successfully achieved by ultrasonic atomization-assisted spray pyrolysis. In as-prepared Si-GR composites, agglomerates of Si nanoparticles were located inside the GR shell, and the composites generally had a crumpled paper ball-like morphology. The average diameter of the composites could be controlled with respect to process variables. Moreover, the composites were employed to fabricate electrode materials for use in a high-capacity lithium-ion battery. The electrochemical performance of the resulting the Si-GR composites showed promise as a high-capacity lithium storage material given its reversible capacity and good capacity retention. The employed aerosol process presented here is very environmentally friendly, as it does not require toxic chemicals to obtain the Si-GR composites and highlights the potential application of silicon sludge waste as a high-capacity anode material in lithium-ion batteries as well. Our results provide a new opportunity to develop highly valuable recycling technology for the fabrication of Si-GR composite anode materials of lithium ion batteries from Si sludge waste.

## Methods

### One step synthesis of Si-GR composite

The Si sludge used in this study mainly consisted of Si particles less than 0.2 μm in diameter generated as kerfs, SiC particles of less than 20 μm from the wafer-slicing saw, and metal impurities from fragments of cutting wire. A mixture of Si/SiC particles with 99.97% purity were prepared from dried Si sludge by HCl (Sigma aldrich, 2 M) treatment for removal of impurities. GO was synthesized by the oxidation of graphite (Alfa Aesar, 99.9%, 74 μm) using a modified Hummer’s method^25^,^26^. In order to prepare the Si-GR composites, aerosol spray pyrolysis (ASP) was conducted from a colloidal mixture solution as a precursor prepared by mixing the as-prepared GO colloid and purified Si/SiC particles. The Si-GR composites were prepared with different operating temperatures (300, 400, 500 °C), concentrations of Si (0.5, 1.0, 2.0 wt%), and concentrations of GO (0.1, 0.2, 0.4 wt%).

**Figure 8:**
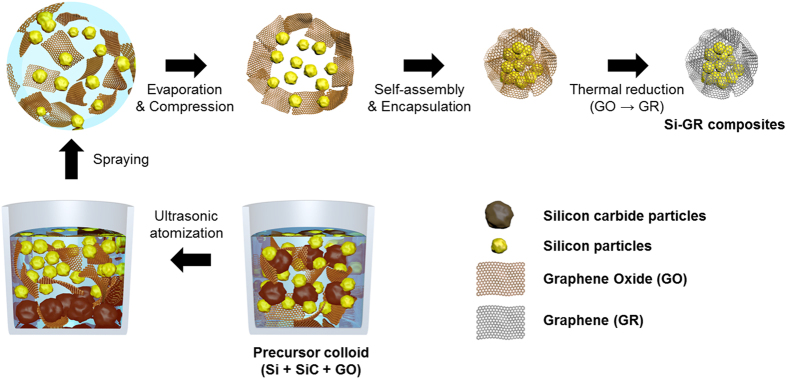
Schematic illustration of the formation of Si-GR composites from colloidal mixture of silicon sludge and graphene oxide via aerosol spray pyrolysis.

A schematic diagram of the synthesis of the Si-GR composites by the ASP is shown in Fig. 8. A colloidal mixture of Si/SiC particles and GO sheets was sprayed by an ultrasonic nebulizer (1.7 MHz, UN-511, Alfesa Pharm Co.). Purified Si/SiC particles were separated into Si and SiC by an ultrasonic atomizer with the result that sprayed Si was carried into a heating zone and then collected in a sampler. Si particles have lighter density and smaller size than SiC particles, so they can be effectively separated by an ultrasonication process in the colloidal solution and fly into a heating zone. At that time, GO sheets were sprayed together with separated Si particles, one droplet containing many GO sheets and Si particles. The evaporation of water in the droplets, a self-assembly process between the Si particles and the GO sheets, and the thermal reduction of the GO to GR were carried out in series in a tubular furnace. The obtained materials compressed each other into micron-sized composites in the shape of a 3D ball. Thus, the Si-GR composites could easily be synthesized by a one-step aerosol process without an additional separation process of Si/SiC or reduction process of GR.

### Materials characterization

The crystallinities of the as-prepared Si-GR composites were analyzed with X-ray diffractometry (XRD; RTP 300 RC, Rigaku, Japan). The morphologies of the as-prepared Si-GR composites were observed with a transmission electron microscope (TEM; JSM-6380LA, JEOL, Japan) and a field emission scanning electron microscopy (FE-SEM; Sirion, FEI, USA). The molecular species of the composite were measured at wavelengths ranging from 1000 to 2000 cm^−1^ with excitation of a 532 nm laser by Raman spectra (Lambda Ray, LSI Dimension P1, Korea). The specific surface area was characterized by analyzing the N_2_ adsorption-desorption isotherms (BET; Tristar 3000, Micromeritics, USA). The thermo gravimetric analysis was conducted to measure the Si content in the composites in a temperature range from 25 to 800 °C with a heating rate of 5 °C/min in air (TGA; DTG-60H, Shimadzu, Japan).

### Electrochemical measurements

Charge/discharge tests were conducted using a CR2032-type coin cell. Metallic lithium was employed as the counter electrode. The working electrode was fabricated by pasting a mixture of the electrode material, carbon black and polyamide imide as a binder (Solvay) at a weight ratio of 80:10:10 onto a copper foil (12 mm diameter) and then compressing this mixture at 10 MPa. The mass loading level was about 0.5 mg per square centimeter of the electrode. The electrode was dried at 120 °C for 2 h in a vacuum and then assembled into a coin cell in an Ar-filled glove box. 1 M LiPF_6_/ethylene carbonate (EC)/dimethyl carbonate (DMC) (1:1 by volume) was used as the electrolyte solution. A microporous polyolefin membrane (Tonen, Japan) was installed as the separator. Galvanostatic charge/discharge measurements were conducted with a TOSCAT3000 (Toyo, Japan) by varying current densities with voltages between 0.005 and 2 V vs Li/Li^+^. Lithium insertion into the Si electrode was referred to as the discharge, and extraction was referred to as the charge. The capacity was determined based on the mass of the electrode materials.

## Additional Information

**How to cite this article**: Kim, S. K. *et al*. One-Step Formation of Silicon-Graphene Composites from Silicon Sludge Waste and Graphene Oxide via Aerosol Process for Lithium Ion Batteries. *Sci. Rep.* **6**, 33688; doi: 10.1038/srep33688 (2016).

## Supplementary Material

Supplementary Information

## Figures and Tables

**Table 1:** Specific surface areas of Si-GR composites at different operating temperatures, Si sludge concentration, and GO concentration.

Operating temperature (°C)	Specific surface area (m^2^/g)	Si sludge concentration (wt%)	Specific surface area (m^2^/g)	GO concentration (wt%)	Specific surface area (m^2^/g)
300	28	0.5	64	0.1	37
400	37	1	37	0.2	46
500	54	2	35	0.4	52

## References

[b1] Green M. A. Silicon solar cells: evolution, high-efficiency design and efficiency enhancements. Semiconductor Sci. Technol. 8, 1–12 (1993).

[b2] van Sark W. G. J. H. M., Brandsen G. W., Fleuster M. & Hekkert M. P. Analysis of the silicon market: will thin films profit. Energ. Policy 35, 3121–3125 (2007).

[b3] Lin Y. C., Wang T. Y., Lan C. W. & Tai C. Y. Recovery of silicon powder from kerf loss slurry by centrifugation. Powder Technol. 200, 216–223 (2010).

[b4] Woditsch P. & Koch W. Solar grade silicon feedstock supply for PV industry. Sol. Energy Mater. Sol. Cells 72, 11–26 (2002).

[b5] Wang T. Y. . Recovery of silicon from kerf loss slurry waste for photovoltaic applications. Prog. Photovolt: Res. Appl. 17, 155–163 (2009).

[b6] Kong M. S., Jung H. C., Hong H. S., Kim G. S. & Chung H. S. A study of hot consolidation properties for recycled silicon powder. Curr. Appl. Phys. 11, S54–S58 (2011).

[b7] Lin Y. C. & Tai C. Y. Recovery of silicon powder from kerfs loss slurry using phase-transfer separation method. Sep. Purif. Technol. 74, 170–177 (2010).

[b8] Zhang L. & Ciftja A. Recycling of solar cell silicon scraps through filtration, Part 1: experimental investigation. Sol. Energ. Mat. Sol. C. 92, 1450–1461 (2008).

[b9] Jang H. D., Kim H., Kil D. S. & Chang H. A novel recovery of silicon nanoparticles from a waste silicon sludge, J. Nanosci. Nanotechnol. 13, 2334–2338 (2013). 23755688 10.1166/jnn.2013.6909

[b10] Jang H. D. . Aerosol-assisted extraction of silicon nanoparticles from wafer slicing waste for lithium ion batteries, Sci. Rep. 5, 9431 (2015). 25819285 10.1038/srep09431 PMC4377548

[b11] Li N., Jin S., Liao Q., Cui H. & Wang C. X. Encapsulated within graphene shell silicon nanoparticles anchored on vertically aligned graphene trees as lithium ion battery anodes. Nano energy 5, 105–115 (2014).

[b12] Liu N. . A yolk-shell design for stabilized and scalable Li-ion battery alloy anodes. Nano Lett. 12, 3315–3321 (2012). 22551164 10.1021/nl3014814

[b13] He Y. . A novel bath lily-like graphene sheet-wrapped nano-Si composite as a high performance anode material for Li-ion batteries. RSC Adv. 1, 958–960 (2011).

[b14] Bao Q., Huang Y., Lan C., Chen B. & Duh J. Scalable upcycling silicon from waste slicing sludge for high-performance lithium-ion battery anodes. Electrochim. Acta. 173, 82–90 (2015).

[b15] Wang D. . Enhanced cycle stability of micro-sized Si/C anode material with low carbon content fabricated via spray drying and *in situ* carbonization. J. Alloy Compd. 604, 130–136 (2014).

[b16] Jung D. S., Hwang T. H., Park S. B. & Choi J. W. Spray drying method for large-scale and high-performance silicon negative electrodes in Li-ion Batteries. Nano Lett. 13, 2092–2097 (2013). 23537321 10.1021/nl400437f

[b17] Li M. . Facile spray-drying/pyrolysis synthesis of core-shell structure graphite/silicon-porous carbon composite as a superior anode for Li-ion batteries. J. Power Sources 248, 721–728 (2014).

[b18] Chabot V. . Graphene wrapped silicon nanocomposites for enhanced electrochemical performance in lithium ion batteries. Electrochim. Acta. 130, 127–134 (2014).

[b19] Luo J. . Crumpled graphene-encapsulated Si nanoparticles for lithium ion battery anodes. J. Phys. Chem. Lett. 3, 1824–1829 (2012). 26291867 10.1021/jz3006892

[b20] Yi R., Zai J., Dai F., Gordin M. L. & Wang D. Dual conductive network-enabled graphene/Si-C composite anode with high areal capacity for lithium-ion batteries. Nano energy. 6, 211–218 (2014).

[b21] Tang H. . Self-assembly silicon/porous reduced graphene oxide composite film as a binder-free and flexible anode for lithium-ion batteries. Electrochim. Acta. 156, 86–93 (2015).

[b22] Lin Y. C., Wang T. Y., Lan C. W. & Tai C. Y. Recovery of silicon powder form kerf loss slurry by centrifugation. Power Technol. 200, 216–223 (2010).

[b23] Yang Y. . Graphene encapsulated and SiC reinforced silicon nanowires as an anode material for lithium ion batteries. nanoscale. 5, 8689–8694 (2013). 23900559 10.1039/c3nr02788k

[b24] Ferrari A. C. . Raman spectrum of graphene and graphene Layer. Phys.Rev. Lett. 97, 187401 (2006). 17155573 10.1103/PhysRevLett.97.187401

[b25] Hummers W. S. & Offeman R. E. Preparation of graphitic oxide. J. Am. Chem. Soc. 80, 1339–1339 (1958).

[b26] Cote L. J., Kim F. & Huang J. Langmuir-blodgett assembly of graphite oxide single layers. J. Am. Chem. Soc. 131, 1043–1049 (2009). 18939796 10.1021/ja806262m

