# Supplementary material for: One-Step Formation of Silicon-Graphene Composites from Silicon Sludge Waste and Graphene Oxide via Aerosol Process for Lithium Ion Batteries
Source: Sci Rep. 2016 Sep 20;6:33688. doi: 10.1038/srep33688 (PMC5029287; doi:10.1038/srep33688)
Supplement: Supplementary Information [file srep33688-s1.doc]

Supplementary Information

**One-Step Formation of Silicon-Graphene Composites from Silicon Sludge Waste and Graphene Oxide via Aerosol Process for Lithium Ion Batteries**

Sun Kyung Kim^1^, Hyekyoung Kim^1,2^, Hankwon Chang^1,2^, Bong-Gyoo Cho^3^, Jiaxing Huang^4,***^, Hyundong Yoo^5^, Hansu Kim^5,**^, Hee Dong Jang^1,2,*^

^1^Rare Metals Research Center, Korea Institute of Geoscience & Mineral Resources, Daejeon, 34132, Korea

^2^Department of Nanomaterials Science and Engineering, University of Science & Technology, Daejeon, 34113, Korea

^3^R&D Center for Valuable Recycling, Korea Institute of Geoscience & Mineral Resources, Daejeon, 34132, Korea

^4^Department of Materials Science and Engineering, Northwestern University, Evanston,

Illinois 60208, USA

^5^Department of Energy Engineering, Hanyang University, Seoul, 04763, Korea


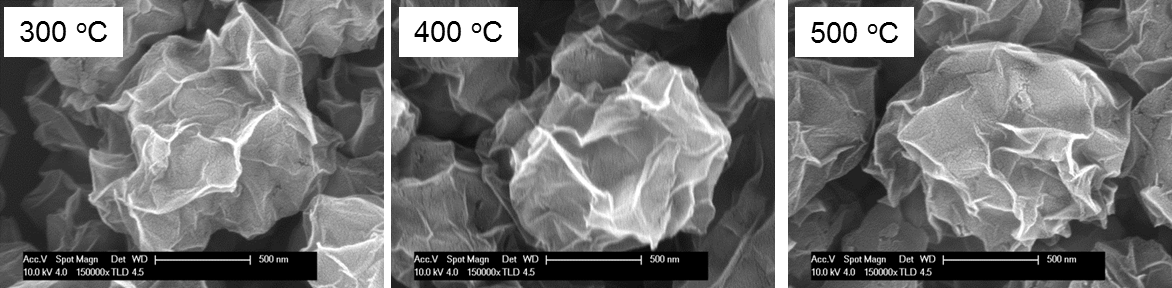


Figure S1 | Morphology of the Si-GR composites prepared at different operating temperatures of 300 ^o^C, 400 ^o^C, and 500 ^o^C (GO: 0.1 wt%, Si sludge: 1.0 wt%).


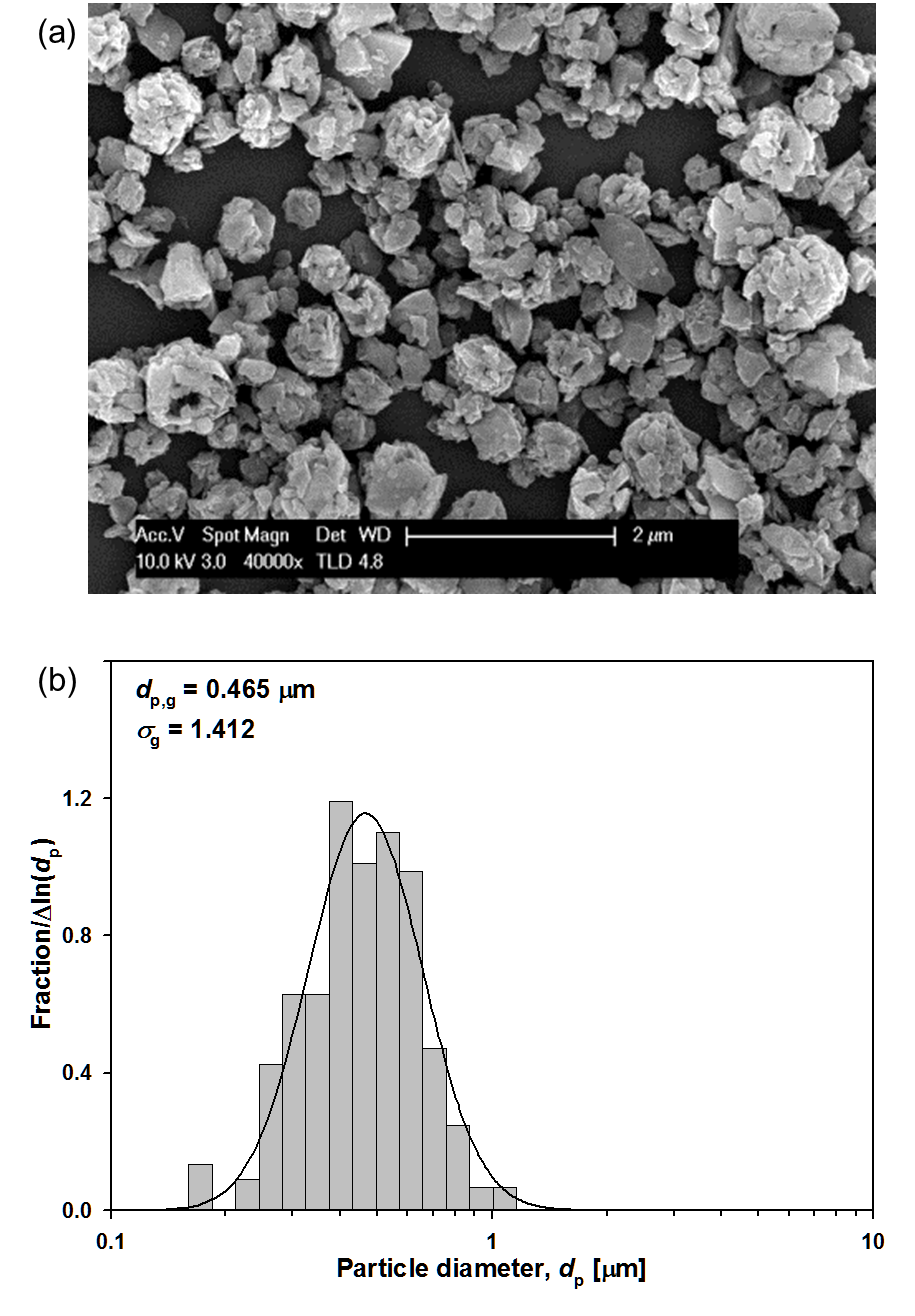


Figure S2 | (a) Morphology, and (b) particle size distribution of the as-fabricated Si agglomerates recovered by ultrasonic atomization from colloidal mixture of Si/SiC (Si sludge: 1.0 wt%, operating temperature: 300 ^o^C).


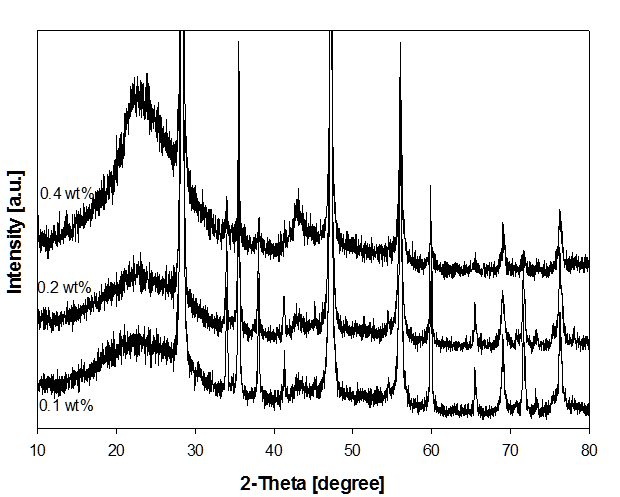


Figure S3 | X-ray diffraction pattern of the Si-GR composites prepared at different GO concentrations of 0.1 wt%, 0.2 wt% and 0.4 wt% (Si sludge: 1.0 wt%, operating temperature: 400 ^o^C).


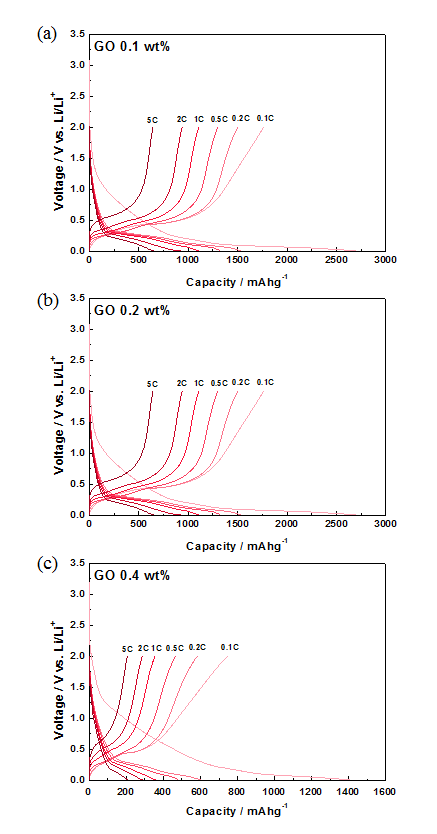


Figure S4 | Rate-capability of Si-GR composites prepared at different GO concentrations of (a) 0.1 wt%, (b) 0.2 wt%, and (c) 0.4 wt% (Si sludge: 1.0 wt%, operating temperature: 400 ^o^C).
